# Development of a core outcome set for studies involving patients undergoing major lower limb amputation for peripheral arterial disease: study protocol for a systematic review and identification of a core outcome set using a Delphi survey

**DOI:** 10.1186/s13063-017-2358-9

**Published:** 2017-12-28

**Authors:** Graeme K. Ambler, David C. Bosanquet, Lucy Brookes-Howell, Emma Thomas-Jones, Cherry-Ann Waldron, Adrian G. K. Edwards, Christopher P. Twine

**Affiliations:** 1 Aneurin Bevan University Health Board, Royal Gwent Hospital, Cardiff Road, Newport, NP20 2UB UK; 2 Division of Population Medicine, Cardiff University, 5th Floor, Neuadd Meirionnydd, Heath Park, Cardiff, CF14 4XW UK; 3 Centre for Trials Research, Cardiff University, 7th Floor, Neuadd Meirionnydd, Heath Park, Cardiff, CF14 4XW UK

**Keywords:** Amputation, Peripheral arterial disease, Vascular surgical procedures, Outcome assessment (Healthcare)

## Abstract

**Background:**

The development of a standardised reporting set is important to ensure that research is directed towards the most important outcomes and that data is comparable. To ensure validity, the set must be agreed by a consensus of stakeholders including patients, healthcare professionals and lay representatives. There is currently no agreed core outcome set for patients undergoing major lower limb amputation for peripheral arterial disease (PAD) for either short- or medium-term research outcomes. By developing these sets we aim to rationalise future trial outcomes, facilitate meta-analysis and improve the quality and applicability of amputation research.

**Methods/design:**

We will undertake a comprehensive systematic review of studies of patients undergoing major lower limb amputation for PAD. Data regarding all primary and secondary outcomes reported in relevant studies will be extracted and summarised as outcome domains. We will then undertake focus groups with key stakeholders (patients, carers, health and social care workers) to collect qualitative data to identify the main short- and medium-term research outcomes for patients undergoing major lower limb amputation. Results of the systematic review and focus groups will be combined to create a comprehensive list of potential key outcomes. Stakeholders (patients, researchers and health and social care workers) will then be polled to determine which of the outcomes are considered to be important in a general context using a three-phase Delphi process. After preliminary analysis, results will be presented at a face-to-face meeting of key stakeholders for discussion and voting on the final set of core outcomes. This project is being run in parallel with a feasibility trial assessing perineural catheters in patients undergoing lower limb amputation (the PLACEMENT trial). Full ethical approval has been granted for the study (Wales REC 3 reference number 16/WA/0353).

**Discussion:**

Core outcome sets will be developed for short- and medium-term outcomes of research involving patients undergoing major lower limb amputation for PAD. This will help with the design of future trials and facilitate meta-analyses of trial data.

**Trial registration:**

PROSPERO: CRD42017059329. Registered on 30 March 2017.

COMET: 975. Registered on 5 April 2017.

**Electronic supplementary material:**

The online version of this article (doi:10.1186/s13063-017-2358-9) contains supplementary material, which is available to authorized users.

## Background

The rising prevalence of diabetes combined with high historical rates of smoking have resulted in global levels of peripheral arterial disease (PAD) exceeding 10% in 65–69 year olds [1]. Despite advances in techniques for revascularisation, a small but significant proportion (1–2%) of these patients will progress to non-reconstructable or non-salvageable PAD, and be faced with major lower limb amputation [2]. This has led to approximately 5000 major lower limb amputations being performed each year in the United Kingdom alone [3]. A recent UK-wide report highlighted the substandard outcomes experienced by these patients; including poor pain control, delays getting patients to the operating room and high rates of in-hospital mortality [2]. Outcomes in the UK appear to be worse than in other developed countries [2]. There is, therefore, an urgent need for research into improving outcomes for patients undergoing lower limb amputation.

Systematic review with meta-analysis is the optimal strategy for pooling results from multiple studies, but it is being increasingly realised that many studies involving similar patient cohorts report similar, but subtly different, outcomes [4]. This heterogeneity makes meta-analysis difficult, and it is often impossible to generate pooled effect estimates [5]. This can result in studies being excluded from analysis simply because their outcomes are not directly comparable. In response to this issue, a growing number of ‘core outcome sets’ have been developed [6]. Core outcome sets aim to find consensus on which key outcomes should be reported for all studies involving a particular group of patients, presenting a minimum standard. If adopted, future research will then be more directly comparable. In addition to this, they aim to reduce research waste by directing research towards the most important outcomes, and reduce the under-reporting of harms by listing the important harms which should be reported in clinical studies.

Although there has been some work examining core outcomes for longer-term functional issues in established amputees [7], there is no consensus about which short- (within 30 days) and medium-term (up to 2 years) outcomes are important to report for patients undergoing major lower limb amputation. These definitions of short-term and medium-term were chosen because many established quality metrics in surgery are concerned with outcomes such as mortality or readmission within 30 days; and after consulting colleagues in rehabilitation, who told us that they would regard patients 2 years after their amputation as ‘established’ amputees. There is currently a significant focus on the poor short- and medium-term outcomes of amputees in the UK [3], so it is vitally important that core outcomes sets for both short- and medium-term outcomes are developed soon. These should then be reported in any study involving patients undergoing major lower limb amputation. The lack of core outcome sets for patients undergoing major lower limb amputation was evident to the authors when designing a randomised controlled feasibility trial examining the use of a perineural catheter to improve pain following major lower limb amputation (Perineural Local Anaesthetic Catheter aftEr Major lowEr limb amputatioN (PLACEMENT) trial) [8]. Development of these core outcome sets will, therefore, be undertaken in tandem with this study (Wales Research Ethics Committee (REC) 3 reference number 16/WA/0353).

The aim of the current work is to develop core outcome sets for short- and medium-term outcomes for research involving patients undergoing major lower limb amputation for complications of peripheral vascular disease. The reason for restricting attention to this subgroup of amputees and excluding those patients undergoing amputation for other reasons, such as trauma or tumour, is that these two subsets of patients are quite distinct. Patients undergoing amputation for complications of peripheral vascular disease are generally older, with significant co-morbidities. Patients undergoing amputation for trauma or tumour are generally younger, and often otherwise healthy. The latter patient group often return to full independence quickly, whereas the former have a significant risk of not even surviving admission, and often have a prolonged, difficult rehabilitation phase. It was, therefore, felt that core outcomes for the two groups might be quite different, so we focussed attention on the larger subset, which in most countries is the subset with peripheral vascular disease.

Drawing upon methods used in the development of previous core outcome sets and described in *The COMET Handbook* [9], the study will take a mixed-methods approach [10], utilising both quantitative and qualitative aspects. It will be undertaken in four key stages as described in the *Handbook*: (1) a systematic review to identify existing published outcomes; (2) focus groups to ensure that published outcomes adequately capture the issues which are most important to patients undergoing amputation as well as those who care for them; (3) a consensus (Delphi) survey; and (4) generation of the final core outcome set using the results of the consensus survey and a nominal group technique. The objective of this report is to describe a protocol for the development of a core outcome set for studies of major lower limb amputation for peripheral vascular disease using this process.

## Methods/design

### Phase I: Systematic review

The first stage of core outcome set development will involve a systematic review of published academic literature. The objective of this phase is to create a long-list of outcome measures which have been reported in previous studies. The review will be conducted according to the Preferred Reporting Items for Systematic Reviews and Meta-Analyses (PRISMA) Statement as appropriate [11], and has been registered in the PROSPERO registry (ID: CRD42017059329).

#### Criteria for considering studies

All clinical studies reporting at least one short- (within 30 days) or medium-term (up to 2 years) outcome involving human subjects undergoing major lower limb amputation (i.e. amputation of the lower limb above the ankle) as a result of PAD will be included. This includes non-interventional studies (e.g. case series, cohort and qualitative studies), non-randomised and randomised interventional trials. Study reports describing the same patient sample will be included if they report different outcomes, but outcomes which are duplicated will only be counted once in any quantification of the frequency of outcome reporting. Studies reporting only patients undergoing amputation for non-ischaemic disease, such as trauma, tumour, chronic non-ischaemic pain or congenital malformations, will be excluded. Systematic reviews will be included as providing a source of additional references which might otherwise be missed. Non-systematic reviews, commentary, editorials and articles which discuss general principles rather than patient cases will be excluded. Non-English language clinical studies will be included if there is a publicly available translation of either the abstract or full study, and data extraction will be limited to what is available in English.

#### Outcomes

All outcomes described as either primary or secondary outcomes from included studies will be reported. When more than a single study reports an outcome, the number and proportion of studies reporting that outcome will be recorded.

#### Search strategy

Medical Literature Analysis and Retrieval System Online (MEDLINE) and Excerpta Medica dataBASE (EMBASE) will be searched through Ovid using the Medical Subject Headings (MeSH) terms given in Additional file 1: Appendix A. Titles will be screened then abstracts of potentially relevant articles will then be retrieved, screened and full-text articles retrieved when necessary to determine inclusion in the study. Reference lists of included studies will also be screened, and a search using the ‘Related Articles’ function in Public MEDLINE interface (PubMed) will capture any further relevant papers. Two individuals will independently screen studies for inclusion. Disagreements will be resolved through discussion and consensus. A flow chart will be presented to describe the search process and results.

#### Data extraction

A standardised data collection proforma will be used. Extracted data will include the participant details (number and demographics: age, gender and study country), study type (for example, randomised or non-randomised controlled trial, cohort study, case series, qualitative), interventions (if any), stated outcomes presented in the methods (both primary and secondary) and reported outcomes. Outcomes will be extracted verbatim. As this study focusses on which outcomes are reported rather than the value of those outcomes, neither study quality nor risk of bias is relevant so will not be assessed. Data will be extracted from 10% of studies by two independent reviewers. Concordance between reviewers will be maximised by discussing in detail the first 10% and coming to a consensus decision. Following this, the next 10% will also be extracted independently and concordance will then be assessed by calculating Kendall’s τ (tau) statistic for the number of extracted outcomes. Provided that the concordance between reviewers is high (i.e. a 95% confidence interval for the value of τ includes zero), the remainder of studies will be extracted by a single reviewer. If concordance is poor, discrepancies will be investigated and a further 10% of studies will be double-extracted. If concordance is high at this point, the remainder of studies will be single-extracted, otherwise double-extraction and consensus will continue.

#### Results synthesis

The principal outcome of the systematic review is a list of outcomes, with frequencies of reporting. Following generation of this (long)-list, outcomes will be grouped by the study authors into appropriate domains in order to draw out common themes for consideration in qualitative focus groups, discussed below. For example ‘30-day mortality’, ‘in-hospital mortality’ and ’12-month mortality’ would all be grouped into the domain ‘mortality’.

### Phase II: Qualitative focus groups

Following the systematic review, we will conduct focus groups with key stakeholders to identify further outcomes not identified in the systematic review. The stakeholders will include patients who have had a major lower limb amputation, family/carers, surgeons, anaesthetists, rehabilitation physicians, nurses, physiotherapists, occupational therapists, prosthetics technicians, social workers and other allied groups affiliated to amputees and their care. If a certain group cannot be represented in a focus group we may interview them on a separate occasion. We anticipate three focus groups, comprising one with patients and carers, one with physicians and one with other healthcare professionals, each with 6–10 participants. This is based on guidance on focus groups in terms of numbers of groups and numbers of participants [12]. While the sample population is relatively small, efforts will be made to include representatives from a range of professions, and patients (in terms of type of amputation, gender). The research team will be pragmatic in their sample size and the need to conduct further focus groups will be based on preliminary analysis/facilitator field notes indicating whether the data collected sufficiently answers the research question [13]. In line with recent methodological debates on the notion of saturation in qualitative research [13], we will maintain transparency in our approach by keeping detailed notes on our sampling strategy. In real terms, decision on whether to sample more participants will be based on discussions within the research team (GA and LBH) about whether there is sufficient breadth and depth of data, whether the specific participants represent the research topic, and practical aspects of recruitment (taking into consideration attempts to invite participants, numbers declined, and withdrawn). Informed consent will be obtained for all participants by trained study personnel.

#### Data collection

We will use a flexible, semi-structured topic guide and begin with an open discussion of issues relating to the care of patients undergoing major lower limb amputation, the level of importance participants place on these issues, and how these issues may change over time (i.e. over the short- and medium-term time periods). The first part of the interview will be guided by participants themselves, and reference to the areas identified in the systematic review will not be revealed. However, after this open discussion, prompts from the outcome domains developed in the systematic review may be used if areas have not naturally occurred. The facilitator will use these prompts to explore whether the outcome domains revealed by the systematic review are relevant to the real-life experiences and attitudes of the focus group participants and whether they are comprehensive to the concerns and needs of patients with lower limb amputations. We will encourage participants to initiate and elaborate on topics most important to them. Participants will be encouraged to respond directly to other participants’ responses in order to generate a group discussion. Focus groups are likely to take around 60-90 min and will be audio-recorded and transcribed verbatim, with references to identifiable personal details removed. Brief demographic details of participants will be taken by the facilitator. Field notes will be made by the facilitator following the focus groups which will include reflections on the process, overall observations, and relevant contextual details. The data will be managed using qualitative coding software (NVivo qualitative analysis software; QSR International Pty Ltd., Version 11). Data will be coded, stored and analysed at the Centre for Trials Research, and kept on encrypted storage devices. The study is coordinated by the CTR, who will monitor and audit study procedures. Monitoring will be conducted independently by a qualified member of CTR staff not participating in the day-to-day study activities.

#### Analysis

We will carry out thematic analysis of the focus group transcripts, and the facilitator’s field notes [14]. Following familiarisation with the data, LBH will develop a way of categorising the data into themes and subthemes (the analytical framework). We will take an inductive approach, where the themes are identified directly from the focus group data, without referring to the categories identified in the systematic review. LBH and GA will discuss the framework and agree a framework between them. LBH will then systematically code the focus group data, using qualitative data analysis software NVivo (QSR International Pty Ltd.), according to these themes (data topics that are common in the dataset), but also looking for contradictory views (negative cases). GA will code a proportion of the dataset independently (10–20%) and LBH and GA will meet to discuss discrepancies in coding until consensus is reached. Any refinements will be made to the analytical framework and reapplied to the data. LBH will then interpret the coded data, taking into consideration the stakeholder group (i.e. themes according to patient, carer, health professional type). The next, and final stage of analysis will then involve considering this interpretation of the focus group data against the outcome domains identified in the systematic review. GA and LBH will identify: (1) areas where themes in the focus group data are similar or correspond to those identified in the systematic review; (2) areas where new themes were initiated by focus group participants but were not found in the systematic review; and (3) areas where themes were found in the systematic review but not present in the focus group data. By bringing these elements together we will produce a list of outcome domains to be taken forward to the consensus study.

As we will use an inductive approach in which the data takes centre stage, the theoretical framework is not predetermined but description will be derived from the data itself. We will take a phenomenological approach to attempt to uncover the meaning of the ‘lived experience’ of groups of individuals – in this case people who have undergone major lower limb amputation and their family, and a range of health professionals involved in the management of patients who have undergone amputation – on the phenomenon (issues or outcomes of importance to patients after undergoing major lower limb amputation). As Tavallaei and Abu (2010: p. 575) [15] describe the major aim of phenomenology is to ‘reduce’ the experience individuals have about a certain phenomenon so that finally the description of the universal essence is created which means “to grasp the very nature of the thing” (VanManen, 1990:177) [16].

### Phase III: Consensus survey

Following synthesis of results from the systematic review and qualitative focus groups, stakeholders (patients and health and social care workers) will be surveyed to determine which outcomes should comprise the core outcome set for studies of lower limb amputation for PAD. The list of stakeholders will include those participating in the focus groups in addition to health and social care workers, who will be invited to contribute via national and international societies, and the corresponding authors of studies included in phase I. This will be a Delphi consensus process [17], and will mainly use an online survey tool, but a paper version will also be available for participants who prefer this. Stakeholders will be asked to rate the putative outcomes on a 1–9 Likert-like scale, with 7–9 labelled as ‘essential’ (must be reported in all trials), 4–6 as ‘desirable’ and 1–3 as ‘not important’. Outcomes not achieving a mean score of greater than 6 by the respondents will be eliminated, and the process will proceed to a second round of voting.

In the second round, stakeholders will again be asked to rate the putative outcomes as essential (7–9), desirable (4–6) or not important (1–3). They will also be given the opportunity to propose outcomes that they feel to be essential but have been excluded from the first round. Any additional outcomes proposed in this way will be considered by the study authors, and added to the list of potential core outcomes for voting. Outcomes not achieving a mean score of at least 7 will be eliminated, and the process will proceed to a third round of voting.

In the third round, stakeholders will be asked to rate the putative outcome measures as essential (7–9), desirable (4–6) or not important (1–3). Outcomes not voted ‘essential’ (7–9) by 75% of the respondents will be eliminated.

At each stage, participants will be asked to rate outcomes separately for short-term and medium-term studies. This is because it is recognised that some outcomes may be considered more or less important depending upon the timing of the study. For example, stakeholders may consider the rate of post-operative pneumonia very important for short-term studies but less important for medium-term studies, whereas the rate of prosthetic limb prescription may be considered very important for medium-term studies but less important for short-term studies.

### Phase IV: Synthesis of results and nominal group analysis

The ultimate goal of this research is to define core sets of short- and medium-term outcomes for reporting by research studies on patients undergoing major lower limb amputation for PAD. The results of the consensus survey will, therefore, be discussed at a face-to-face meeting of key stakeholders and a nominal group technique applied to determine a list of short-term outcomes and a second list of medium-term outcomes which will represent the core outcome sets. Stakeholders will include members of the PLACEMENT Trial Management Group, along with individuals from professions or specialties not represented by the Group, who participated in the focus groups in phase II. A nominal group technique will be used rather than a straightforward vote to either accept or reject the results of the consensus survey because of the risk that by choosing somewhat arbitrary levels at which to eliminate outcomes during the Delphi process, it is possible to arrive at either a core outcome set with an enormous number of items, or a core outcome set with only a very small number of items. By using a nominal group technique, the members of the face-to-face meeting, therefore, have the opportunity to present potential solutions to these problems if they arise, rather than simply voting to reject the result of the Delphi.

### Publication and dissemination of results

All publications and presentations relating to the study will be authorised by the PLACEMENT Trial Management Group and will be in accordance with the main trial’s publication policy. In addition to the required final report and monograph for the funding body, we will publish the main study results in international, peer-reviewed, open-access journals and present them at national and international scientific meetings. With the assistance of our collaborators and lay representatives we will disseminate the trial findings to a wide audience and vigorously promote uptake of the trial results into clinical care. This will include presentations at meetings and written executive summaries for key stakeholder groups such as Secondary Care Trusts, Royal Colleges, Medical Schools and relevant patient groups. Access to the full protocol, anonymised participant-level data, and statistical code will be available from the study team upon request after the main study results have been published.

## Discussion

Short- and medium-term outcomes after major lower limb amputation are surprisingly poor, especially in the UK [2]. Given that the global diabetes epidemic has caused rates of PAD to rise by over a third in the first decade of the 21st century [1], it is likely that major lower limb amputation rates will also rise. There is, therefore, an urgent need for high-quality research to improve these outcomes. Core outcome sets for both short- and medium-term outcomes are critical so that trials can concentrate on the most important issues to patients and healthcare workers in a way that facilitates future meta-analysis and guideline development.

## Study status

The systematic review is currently underway.

## Additional files

Additional file 1: Appendix A Search Details. (DOCX 85 kb)

## Abbreviations

EMBASE: Excerpta Medica dataBASE
MEDLINE: Medical Literature Analysis and Retrieval System Online
MeSH: Medical Subject Headings
MINORS: Methodological Index for Non-randomized Studies
PAD: Peripheral arterial disease
PLACEMENT: Perineural Local Anaesthetic Catheter aftEr Major lowEr limb amputatioN Trial
PRISMA: Preferred Reporting Items for Systematic Reviews and Meta-Analyses
PubMed: Public MEDLINE interface
REC: Research Ethics Committee
