# Supplementary material for: Development of a core outcome set for studies involving patients undergoing major lower limb amputation for peripheral arterial disease: study protocol for a systematic review and identification of a core outcome set using a Delphi survey
Source: Trials. 2017 Dec 28;18:628. doi: 10.1186/s13063-017-2358-9 (PMC5747272; doi:10.1186/s13063-017-2358-9)
Supplement: Supplementary file 1 — Appendix A Search Details. (DOCX 85 kb) [file 13063_2017_2358_MOESM1_ESM.docx]

# Appendix A – Search Details

Below is the list of MeSH terms used for searching MEDLINE and EMBASE. This search was performed via Ovid using the following search criteria on 13^th^ March 2017 and the numbers of results for each item are given.

| **#** | **Search item** | **Number of results** |
| --- | --- | --- |
| 1 | exp Peripheral Vascular Diseases/di [Diagnosis] | 218253 |
| 2 | Arterial Occlusive Diseases/di [Diagnosis] | 6766 |
| 3 | exp Arteriosclerosis/di [Diagnosis] | 35693 |
| 4 | exp Atherosclerosis/di [Diagnosis] | 18165 |
| 5 | exp Peripheral Arterial Disease/di [Diagnosis] | 22699 |
| 6 | (atherosclero* or arteriosclero* or PVD or PAOD or PAD).ti,ab. | 385166 |
| 7 | (arter$ adj4 ($occlus$ or steno$ or obstruct$ or lesio$ or block$ or obliter$)).ti,ab. | 233846 |
| 8 | (vascular adj4 (occlus* or steno* or obstruct* or lesio* or block* or obliter*)).ti,ab. | 62629 |
| 9 | (vein* adj4 (occlus* or steno* or obstruct* or lesio* or block* or obliter*)).ti,ab. | 30643 |
| 10 | (veno* adj4 (occlus* or steno* or obstruct* or lesio* or block* or obliter*)).ti,ab. | 34079 |
| 11 | (peripher* adj4 (occlus* or steno* or obstruct* or lesio* or block* or obliter*)).ti,ab. | 39183 |
| 12 | (peripheral adj3 dis*).ti,ab. | 85949 |
| 13 | arteriopathic.ti,ab. | 462 |
| 14 | CLI.ti,ab. | 4890 |
| 15 | dysvascular*.ti,ab. | 405 |
| 16 | (leg adj4 (obstruct* or occlus* or steno* or block* or obliter*)).ti,ab. | 1394 |
| 17 | (limb adj4 (obstruct* or occlus* or steno* or block* or obliter*)).ti,ab. | 4192 |
| 18 | (lower adj3 extrem* adj4 (obstruct* or occlus* or steno* or block* or obliter*)).ti,ab. | 3557 |
| 19 | 1-18 combined with OR | 966305 |
| 20 | amputee$.tw. | 11024 |
| 21 | Amputees/ | 44444 |
| 22 | (knee adj3 (disarticulat$ or exarticulat$)).tw. | 538 |
| 23 | (amputat$ adj3 (transfemoral or transtibial or lower limb or lower extremity or above knee or below knee or through knee)).tw. | 11473 |
| 24 | Disarticulation/ | 446 |
| 25 | Amputation/ | 50788 |
| 26 | 20-25 combined with OR | 100428 |
| 27 | (transfemoral or transtibial or lower limb or lower extremity or knee).tw. | 359281 |
| 28 | exp Leg/ | 391890 |
| 29 | 27 OR 28 | 610534 |
| 30 | 26 AND 29 | 28531 |
| 31 | 19 AND 30 | 5849 |
| 32 | Remove duplicates from 31 | 4288 |
